# The complete mitochondrial genome of *Holothuria atra* (Holothuriida: Holothuriidae: *Holothuria*) and its structural characteristics

**DOI:** 10.1371/journal.pone.0347494

**Published:** 2026-04-17

**Authors:** Jinjin Wang, Jianlong Ge, Meijie Liao, Bin Li, Xiaojun Rong, Yingeng Wang, Aijun Cui, Xiaodong Bian, Mingliang Sun, Qingyu Zhang

**Affiliations:** 1 State Key Laboratory of Mariculture Biobreeding and Sustainable Goods, Yellow Sea Fisheries Research Institute, Chinese Academy of Fishery Sciences, Qingdao, PR China; 2 Laboratory for Marine Fisheries Science and Food Production Processes, Laoshan Laboratory, Qingdao, PR China; 3 Zhejiang Ocean Family Co., Ltd, Hangzhou, PR China; National Cheng Kung University, TAIWAN

## Abstract

Sea cucumbers, as typical representatives of deep-sea benthic animals, possess significant scientific and economic importance. This investigation examined the genomic structural characteristics and genetic evolutionary relationships of *Holothuria atra* through the analysis of its complete mitochondrial genome. The findings indicated that the total length of the mitochondrial genome of the *H. atra* specimen was 15,788 bp, encompassing seven NADH genes, three cox genes, two ATP genes, and one cob gene. Additionally, two rRNAs and 22 tRNAs were identified. The entire sequence contained a total of 18 non-coding regions and seven overlapping gene regions, with the combined A + T content reaching 59.2%. The lengths of the 22 tRNAs ranged from 62 to 72 base pairs, and their cloverleaf secondary structures were predicted. Regarding codon usage, the PCGs within the mitochondrial genome of *H. atra* utilized 61 codons, encoding information for 20 amino acids. The most abundantly encoded amino acid in the mitochondrial genome of *H. atra* is leucine (Leu), representing 16.58%, while cysteine (Cys) is the least represented, accounting for 1.03%. Codons with higher usage frequencies include AGA (Ser 1), CUA (Leu 1), and CCA (Pro), whereas those with comparatively lower frequencies are GCG (Ala), CCG (Pro), and AGG (Ser 1). Evolutionary analysis revealed that *H. atra* is most closely related to *Holothuria polii*. By comparing the mitochondrial genomic sequences of 19 species within the class Holothuroidea, it was observed that eight mitochondrial sequences are shared among these species. This study provides valuable data supporting genetic evolutionary research and the development and utilization of *H. atra* resources within the Kiribati region.

## 1. Introduction

Sea cucumbers are marine animals classified within the class Holothuroidea of the phylum Echinodermata, with approximately 1,400 species identified globally [1]. These organisms are significant not only as marine food sources but also as valuable medicinal resources [2]. The nutritional and therapeutic advantages of sea cucumbers stem from their rich content of bioactive substances, which include carotenoids, vitamins, fatty acids, gelatin, amino acids, bioactive peptides, minerals, collagen, chondroitin sulfate, and fucoidan, among others [3,4]. *Holothuria atra*, a member of the phylum Echinodermata, Holothuroidea, and *Holothuria*, is an edible species predominantly found in coral reef environments and sandy substrates characterized by calm waters, abundant seagrass, and high organic matter content. This species is widely distributed throughout the Indian-West Pacific region [5,6].

Mitochondrial DNA (mtDNA) is characterized by strict maternal inheritance and a simple structure, small molecular weight, lack of tissue specificity, and a rapid evolutionary rate. It is widely utilized in studies concerning species origin, genetic differentiation, phylogenetic relationships both within and between species, and species identification [7]. Analyzing mtDNA not only serves as a routine method for investigating genetic diversity in species populations but has also emerged as a significant focus of research in evolutionary biology, genomics, and bioinformatics. The complete mitochondrial genome provides valuable information on individual genes and encompasses broad genomic features that are essential for studying genome evolution, phylogenetic relationships, and species identification [8–10]. Moreover, characteristics such as ribosomal RNA (rRNA), transfer RNA (tRNA), gene arrangement, and protein-coding genes (PCGs) in animal mitochondrial genomes are critical topics in the exploration of gene function and genetic variation [11,12].

In recent years, the complete mitochondrial genomes of several sea cucumber species, including *Bohadschia argus*, *Holothuria polii*, and *Benthodytes marianensis*, have been sequenced and assembled [13–15]. Furthermore, the complete genome sequencing and assembly of *Apostichopus japonicus*, *Chiridota heheva*, and *Stichopus monotuberculatus* have been accomplished, which elucidates the functions of various genes and the underlying genetic mechanisms [16–18]. However, research on *H. atra* has primarily concentrated on its nutritional components and bioactive substances, with limited studies addressing its mitochondrial genome or population genetic diversity [19,20]. Therefore, this study utilized high-throughput sequencing technology to assemble the mitochondrial genome of *H. atra* and conducted analyses of its base composition, codon usage patterns, gene arrangement, phylogenetic relationships, and population genetic diversity. These findings provide valuable scientific data to support genetic research and conservation efforts for *H. atra* populations in the Kiribati region.

## 2. Materials and methods

### 2.1 Sample source

A total of 30 Wild *H. atra* specimens were collected from the sea area of Kiribati (1°22’ N, 173°8’ E). The sampling was conducted with the permission of The Fisheries and Marine Resources Development Department of The Republic of Kiribati. The length of the collected individuals was 24.32 ± 6.12 cm, the width was 6.66 ± 0.63 cm, and the weight was 182.50 ± 102.58 g. The sex ratio of distinguishable individuals was 11 females to 2 males. Following their capture from the natural habitat, the specimens were transported to the laboratory, where longitudinal muscle tissues were meticulously dissected and preserved in absolute ethanol for further analysis.

### 2.2 DNA sequencing and genome assembly

Total genomic DNA was extracted from the longitudinal muscle tissue using the DNeasy tissue kit (Qiagen, Beijing, China), adhering to the manufacturer’s protocols. Following DNA isolation, 1 μg of purified DNA was fragmented to 500 bp using the Covaris M220 system, which was employed to construct short-insert libraries as per the manufacturer’s instructions (TruSeq™ Nano DNA Sample Prep Kit, Illumina) and subsequently sequenced on an Illumina NovaSeq 6000 platform (BIOZERON Co., Ltd, Shanghai, China) with 150 bp paired-end reads. Before assembly, raw reads were filtered using Trimmomatic 0.39 (http://www.usadellab.org/cms/index.php?page=trimmomatic). This filtering step aimed to eliminate reads with adaptors, those exhibiting a quality score below 20 (Q < 20), reads containing uncalled bases (“N” characters) at a percentage equal to or greater than 10%, and duplicated sequences. The mitochondrial genome was reconstructed through a combination of de novo and reference-guided assemblies, employing the following three steps. First, the filtered reads were assembled into contigs using MitoZ v2.3, and potential mitochondrial contigs were extracted by aligning against the NCBI mitochondrial genome database. Second, the potential mitochondrial contigs were aligned to the reference mitogenomes using BLAST v2.8.1, with aligned contigs (>Q80% query coverage) being ordered and connected manually according to the reference mitogenomes. GetOrganelle v1.7.5 (https://github.com/Kinggerm/GetOrganelle) was utilized for mitochondrial genome assembly. Finally, MUMmer 3.23 was employed to verify the circularity of these contigs. Through the aforementioned assembly steps, we successfully obtained a circular representation of the *H. atra* mitogenome.

### 2.3 Gene annotation

The mitochondrial genes were annotated using the online tool MITOS2 (http://mitos.bioinf.uni-leipzig.de/index.py). For the prediction of PCGs, tRNA genes, and rRNA genes, the mitochondrial selection “codon Table 9 of Echinoderms and flatworms” (S1 Table) was utilized. The positions of each coding gene were determined through BLAST searches against reference mitochondrial genes. Manual corrections for start and stop codons were conducted using SnapGene Viewer. The circular mitochondrial genome map of *H. atra* was generated using CGview (http://stothard.afns.ualberta.ca/cgview_server/).

### 2.4 Mitochondrial genomic structure analysis

The base composition statistics of 13 PCGs, two rRNAs, and 22 tRNAs were conducted using MEGA 7.0 software [21]. Base usage bias was determined with the equation (1) and equation (2) [22]:


$$\text{AT}-\text{skew }=\frac{A-T}{A+T}$$ (1)

$$\text{GC}-\text{skew }=\frac{G-C}{G+C}$$ (2)

MITOS2 and Vinna(http://rna.tbi.univie.ac.at/forna/) were used for tRNA secondary structure prediction and visual image construction. CodonW v1.4.2 (https://codonw.sourceforge.net/) was used to analyze the codon usage of mitochondrial PCGs by calculating Relative synonymous codon usage (RSCU) values.

### 2.5 Genetic diversity analysis

The 16S rDNA and COⅠ genes were amplified using a Taq PCR master mix kit and two primer sets: 16Sar (5′-CGCCTGTTTATCAAAAACAT −3′) and 16Sbr (5′-CTCCGGTTTGAACTCAGATCA −3′) [23], as well as COⅠ ef (5′-ATAATGATAGGAGGRTTTGG −3′) and COⅠ er (5′-GCTCGTGTRTCTACRTCCAT −3′) [24]. Thermocycling conditions were 95 °C for 60 s; 35 cycles at 95 ℃ for 30 s, 52 °℃ for 30 s, and 72 ℃ for 1 min; and a final extension at 72 °C for 10 min. The amplified products were subjected to bidirectional sequencing after being qualified by 1.5% agarose gel electrophoresis. The variation in the COⅠ region and the 16S rRNA region was analyzed using DnaSP 5.0. This analysis included the number of haplotypes (*H*), haplotype diversity (*H*_*d*_), number of polymorphic sites (*S*), average number of nucleotide differences (k), nucleotide diversity (*P*_*i*_), and distances within groups.

### 2.6 Phylogenetic analysis

Maximum likelihood phylogenetics were constructed using MEGA 7.0 to determine the phylogenetic relationships among species. Tree confidence levels were assessed through 1,000 bootstrap random replicates and data resampling, while distances between all haplotypes were calculated using the General Time Reversible model. As in the Mega software, the General Time Reversible model is one of the options in the ML phylogenetic analysis, and it belongs to the category of the ML phylogenetic analysis. To conduct a more scientific evolutionary analysis of the species in the Holothuroidea, we selected all the publicly available species of the Holothuroidea from NCBI. Each species uses its complete genome to construct a phylogenetic tree for systematic evolution. All mitochondrial genomic reference sequences utilized for comparison were sourced from the NCBI database: *Holothuria hilla* (MN1630001.1), *Holothuria leucospilota* (NC_046849.1), *Holothuria pervicax* (NC_045853.1), *Holothuria spinifera* (NC_046508.1), *Holothuria edulis* (NC_051928.1), *Holothuria polii* (LR694133.1), *Holothuria scabra* (NC_027086.1), *Holothuria forskali* (NC_013884.1), *Holothuria sanctori* (OZ199603.1), *Bohadschia argus* (NC_061402.1), *Holothuria fuscogilva* (MZ305460.1), *Actinopyga echinites* (MN793975.1), *Actinopyga Lecanora* (MW248463.1), *Apostichopus japonicus* (NC_012616.1), *Cucumaria miniata* (AY182376.1), *Stichopus horrens* (MN128376.1), *Stichopus chloronotus* (MZ052220.1), *Colochirus quadrangularis* (MW218895.1), *Euapta godeffroyi* (LC704718.1), *Cercodemas anceps* (NC_054245.1), *Thelenota ananas* (NC_059759.1).

### 2.7 Mitochondrial gene order and rearrangements analysis

Eighteen species of Holothuroidea (Holothuridae: *A. echinites, A. lecanora, B. argus, H. scabra, H. fuscogilva, H. hilla, H. pervicax, H. leucospilota, H. edulis, H. forskali*; Stichopodidae: *A. japonicus, S. chloronotus, S. horrens, T. ananas;* Cucumariidae: *C. quadrangularis, C. anceps, C. miniata;* Synaptidae: *E. godeffroyi)* were selected from the NCBI database, alongside one species from this study, all of which have comprehensive annotations of their mitochondrial gene compositions. A comparative analysis of the mitochondrial gene arrangement order was performed. The species were categorized based on their taxonomic classification and gene arrangement order at the family level, and a comparative diagram illustrating the mitochondrial gene arrangement orders for the selected species was constructed.

## 3. Results

### 3.1 Mitochondrial genome organization

**Fig 1:**
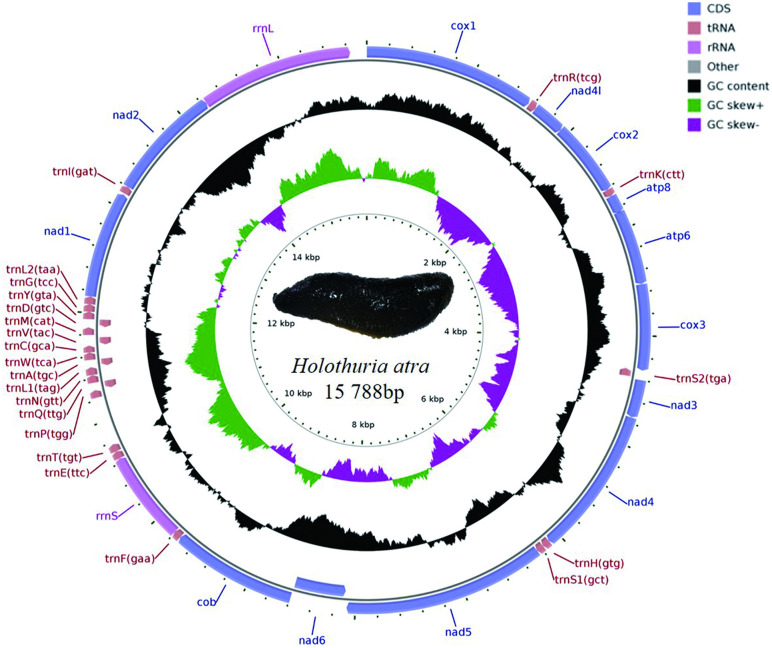
*H. atra* mitogenome gene map.

After sequencing with the Illumina NovaSeq 6000, the raw sequencing data volume obtained was 5,066.3 Mb. Following quality control and trimming, 4,946.9 Mb of clean data was acquired, with a Q20 score of 97.70%. Data assembly yielded 15,788 bp of genomic data. The annotation results for the mitochondrial genome of *H. atra* are presented in Table 1 and Fig 1. The mitochondrial genome sequence and its annotation information have been deposited in GenBank under accession number PV998923. The genes within the genome are closely arranged, with a partial base overlap observed between them. This genome contains seven genes encoding different subunits of NADH dehydrogenase, three genes for different subunits of cytochrome C oxidase, two genes for ATP synthase, one gene for cytochrome B, two rRNAs, and 22 tRNAs. Among the 13 PCGs identified, 12 are situated on the positive strand of the mitochondrial genome, while only one gene, nad6, is positioned on the negative strand. Of the 22 tRNAs, five are located on the negative strand: trnS2, trnQ, trnA, trnV, and trnD. The remaining 17 tRNAs are found on the positive strand. Notably, there are variations in the base composition of different mitochondrial genomes (Table 2). The AT content across various genes ranges from 27.9% to 67.1%, with the highest percentage, 67.1%, observed in trnT and the lowest, 20.6%, in atp8. The overall AT content of the mitochondrial genome is 59.2%. The AT skew across different genes varies from −0.530 to 0.333, with nad6 exhibiting the lowest value of −0.530 and atp8 displaying the highest value of 0.333. The AT skew for the entire mitochondrial genome is 0.154. The GC skew across different genes ranges from −0.524 to 0.400, with atp8 showing the lowest value of −0.524, nad6 presenting the highest value of 0.333, and the overall GC skew of the mitochondrial genome at −0.268.

**Table 1:** Gene characteristics of the *H. atra* mitochondrial genome.

Gene	Position (Start-End)	Length (bp)	Start codon	Stop codon	Anticodon	Space/overlap	Strand
cox1	1-1557	1557	ATG	TAA	–	–	+
trnR	1567-1635	69	–	–	UCG	9	+
nad4l	1636-1932	297	ATG	TAA	–	0	+
cox2	1933-2620	688	ATG	T	–	0	+
trnK	2621-2686	66	–	–	CUU	0	+
atp8	2687-2851	165	ATG	TAA	–	0	+
atp6	2845-3528	684	ATG	TAA	–	−7	+
cox3	3531-4313	783	ATG	TAA	–	2	+
trnS2	4312-4382	71	–	–	UGA	−2	–
nad3	4401-4745	345	ATG	TAA	–	18	+
nad4	4749-6105	1357	ATG	T	–	3	+
trnH	6107-6174	68	–	–	GUG	1	+
trnS1	6176-6243	68	–	–	GCU	1	+
nad5	6244-8079	1836	ATG	TAA	–	0	+
nad6	8097-8585	489	ATG	TAG	–	17	–
cob	8594-9736	1143	ATG	TAA	–	8	+
trnF	9738-9808	71	–	–	GAA	1	+
rrnS	9808-10639	832	–	–	–	−1	+
trnE	10641-10709	69	–	–	UUC	1	+
trnT	10711-10780	70	–	–	UGU	1	+
trnP	11227-11295	69	–	–	UGG	446	+
trnQ	11292-11361	70	–	–	UUG	−4	–
trnN	11364-11433	70	–	–	GUU	2	+
trnL1	11435-11506	72	–	–	UAG	1	+
trnA	11506-11572	67	–	–	UGC	−1	–
trnW	11573-11640	68	–	–	UCA	0	+
trnC	11641-11702	62	–	–	GCA	0	+
trnV	11706-11775	70	–	–	UAC	3	–
trnM	11799-11867	69	–	–	CAU	23	+
trnD	11872-11941	70	–	–	GUC	4	–
trnY	11942-12009	68	–	–	GUA	0	+
trnG	12009-12077	69	–	–	UCC	−1	+
trnL2	12077-12147	71	–	–	UAA	−1	+
nad1	12148-13119	972	ATG	TAA	–	0	+
trnL	13133-13200	68	–	–	GAU	13	+
nad2	13201-14244	1044	ATG	TAA	–	0	+
rrnL	14245-15637	1393	–	–	–	0	+

**Table 2:** Mitochondrial gene nucleotide composition and base bias.

Gene	T%	C%	A%	G%	A + T%	AT-Skew	GC-Skew
cox1	24.8	27.0	30.8	17.4	24.8	0.109	−0.216
nad4l	27.9	31.6	29.6	10.8	27.9	0.029	−0.492
cox2	23.1	28.8	34.2	14.0	23.1	0.193	−0.347
atp8	20.6	29.1	41.2	9.1	20.6	0.333	−0.524
atp6	24.7	31.4	32.5	11.4	24.7	0.136	−0.468
cox3	23.5	29.8	30.8	16.0	23.5	0.134	−0.302
nad3	24.9	30.4	31.0	13.6	24.9	0.109	−0.382
nad4	23.7	27.9	35.2	13.3	23.7	0.196	−0.355
nad5	25.4	25.5	36.0	13.1	25.4	0.172	−0.322
nad6	49.9	10.4	15.3	24.3	49.9	−0.530	0.400
cob	27.4	26.0	32.0	14.6	27.4	0.078	−0.280
nad1	28.3	25.6	31.3	14.8	28.3	0.050	−0.267
nad2	30.4	23.9	33.0	12.8	30.4	0.041	−0.300
rrnS	20.0	23.2	37.5	19.4	57.5	0.305	−0.090
rrnL	20.9	22.4	38.5	18.2	59.4	0.296	−0.102
trnR1	27.5	24.6	34.8	13.0	62.3	0.116	−0.308
trnK	22.7	25.8	34.8	16.7	57.6	0.211	−0.214
trnS2	36.6	14.1	23.9	25.4	60.6	−0.209	0.286
trnH	25.0	20.6	33.8	20.6	58.8	0.150	0.000
trnS1	23.5	25.0	23.5	27.9	47.1	0.000	0.056
trnF	26.8	18.3	33.8	21.1	60.6	0.116	0.071
trnE	20.3	24.6	33.3	21.7	53.6	0.243	−0.063
trnT	30.0	17.1	37.1	15.7	67.1	0.106	−0.043
trnP	23.2	17.4	37.7	21.7	60.9	0.238	0.111
trnQ	35.7	14.3	28.6	21.4	64.3	−0.111	0.200
trnN	25.7	17.1	35.7	21.4	61.4	0.163	0.111
trnL1	33.3	16.7	29.2	20.8	62.5	−0.067	0.111
trnA	37.3	13.4	25.4	23.9	62.7	−0.190	0.280
trnW	29.4	14.7	39.7	16.2	69.1	0.149	0.048
trnC	24.2	24.2	30.6	21.0	54.8	0.118	−0.071
trnV	32.9	18.6	22.9	25.7	55.7	−0.179	0.161
trnM	26.1	24.6	33.3	15.9	59.4	0.122	−0.214
trnD	32.9	15.7	31.4	20.0	64.3	−0.022	0.120
trnY	25.0	22.1	29.4	23.5	54.4	0.081	0.032
trnG	24.6	23.2	36.2	15.9	60.9	0.190	−0.185
trnL2	25.4	25.4	26.8	22.5	52.1	0.027	−0.059
trnI	23.5	26.5	29.4	20.6	52.9	0.111	−0.125
mitogenome	25.0	25.9	34.1	14.9	59.2	0.154	−0.268

### 3.2 Spacers and overlaps

Several adjacent genes exhibited overlapping nucleotides and intergenic spacers within the *H. atra* mitogenome. A total of 18 intergenic spacers, ranging from 1 to 446 bp in size, were identified (Table 1). The largest intergenic spacer, measuring 446 bp, was located between the trnT and trnP genes. Additionally, seven overlaps were noted, with the longest measuring 7 bp, found between the atp8 and atp6 genes. The *H. atra* mitogenome displayed four distinct types of overlaps. The first type involved overlaps between genes located on the “ + ” and “-” strands, with three overlaps identified: 2 bp between trnS2 and cox3, 4 bp between trnP and trnQ, and 1 bp between trnL1 and trnA. The second type of overlap pertained to TA-termination codons, where a 2 bp overlap was observed in the termination codons of cox3. The third type was found within PCGs sequences, specifically a 7 bp overlap between atp6 and cox3. The fourth type of overlap lacked distinct characteristics.

### 3.3 Transfer RNA and ribosomal RNA genes

**Fig 2:**
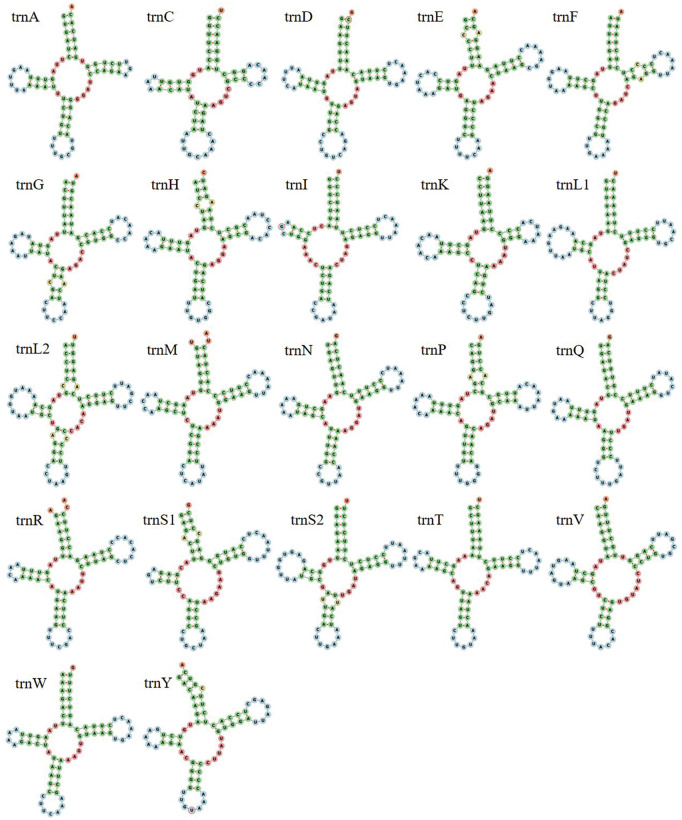
The secondary structure of *H. atra* mitochondrial tRNA.

A total of 22 tRNAs, ranging from 62 to 72 bp in length, were identified within the mitogenome of *H. atra* (Table 1). The longest tRNA is trnL1, while trnC is the shortest. Notably, the *H. atra* mitogenome contains two copies each of trnL and trnS. All tRNAs exhibit the standard cloverleaf secondary structure; however, there are ten pairs of base mismatches, including six pairs on the amino acid acceptor arm, one pair on the TψC arm, and one pair on the anticodon arm (Fig 2). The *H. atra* mitogenome includes two rRNAs. The rrnS, which is 832 bp in length, is located between the trnF and trnE genes. The rrnL, measuring 1,393 bp, is situated between the nad2 and cox1 genes (Table 1).

### 3.4 Codon usage and sequence features of PCGs

The features of the mitochondrial PCGs are summarized in Table 1, which includes gene length, start and stop codons, and their respective strands. All mitochondrial PCGs initiate with an ATG codon. The termination codon TAA is frequently observed. TAG serves as a termination codon for the nad6 gene. The nad5 gene, located between trnS and nad6, is the longest among the 13 PCGs, measuring 1,836 bp. In contrast, the shortest gene, atp8, is situated between trnK and atp6, with a length of 165 bp.

**Fig 3:**
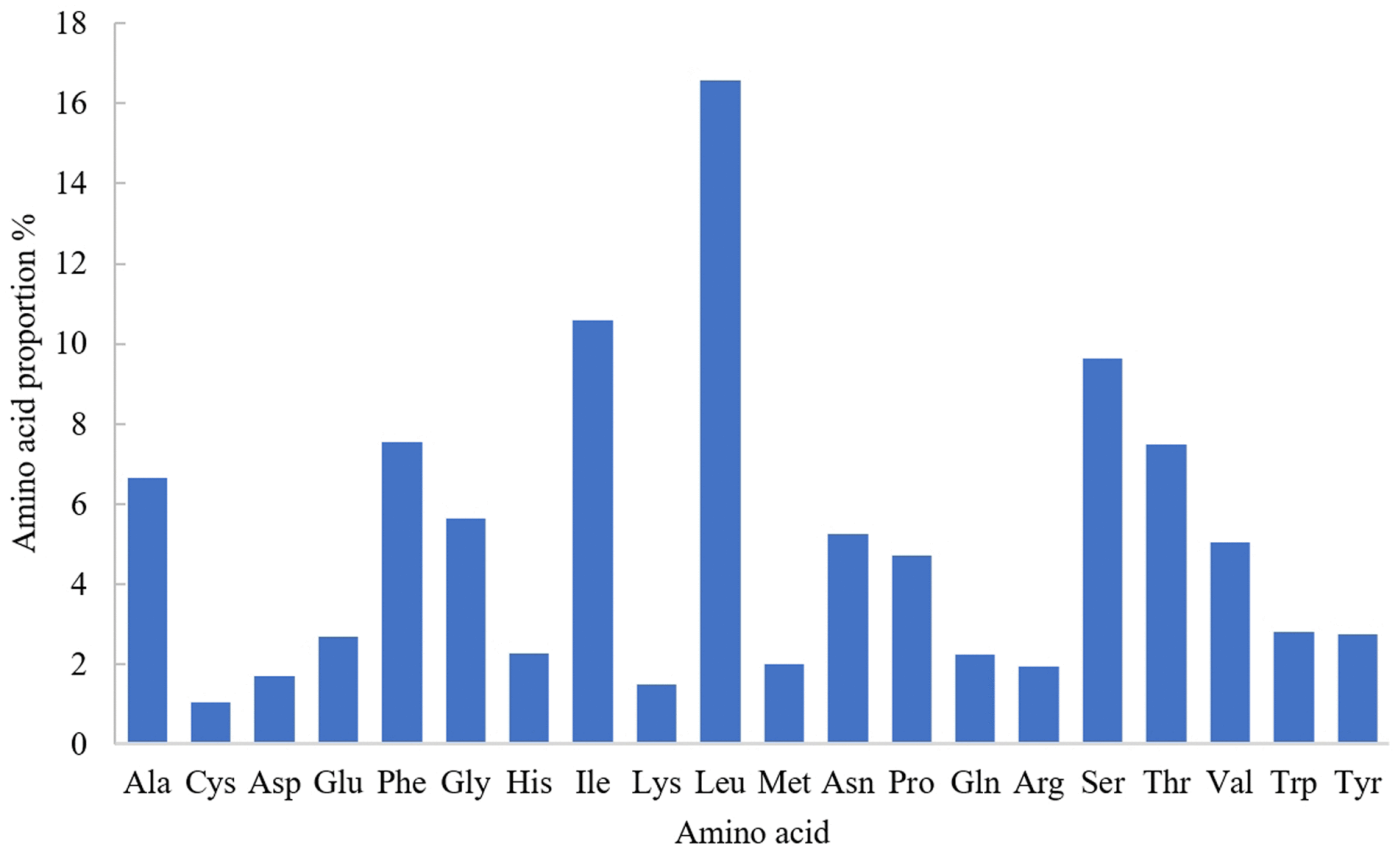
Amino acid composition of mitochondrial gene of *H. atra.*

**Fig 4:**
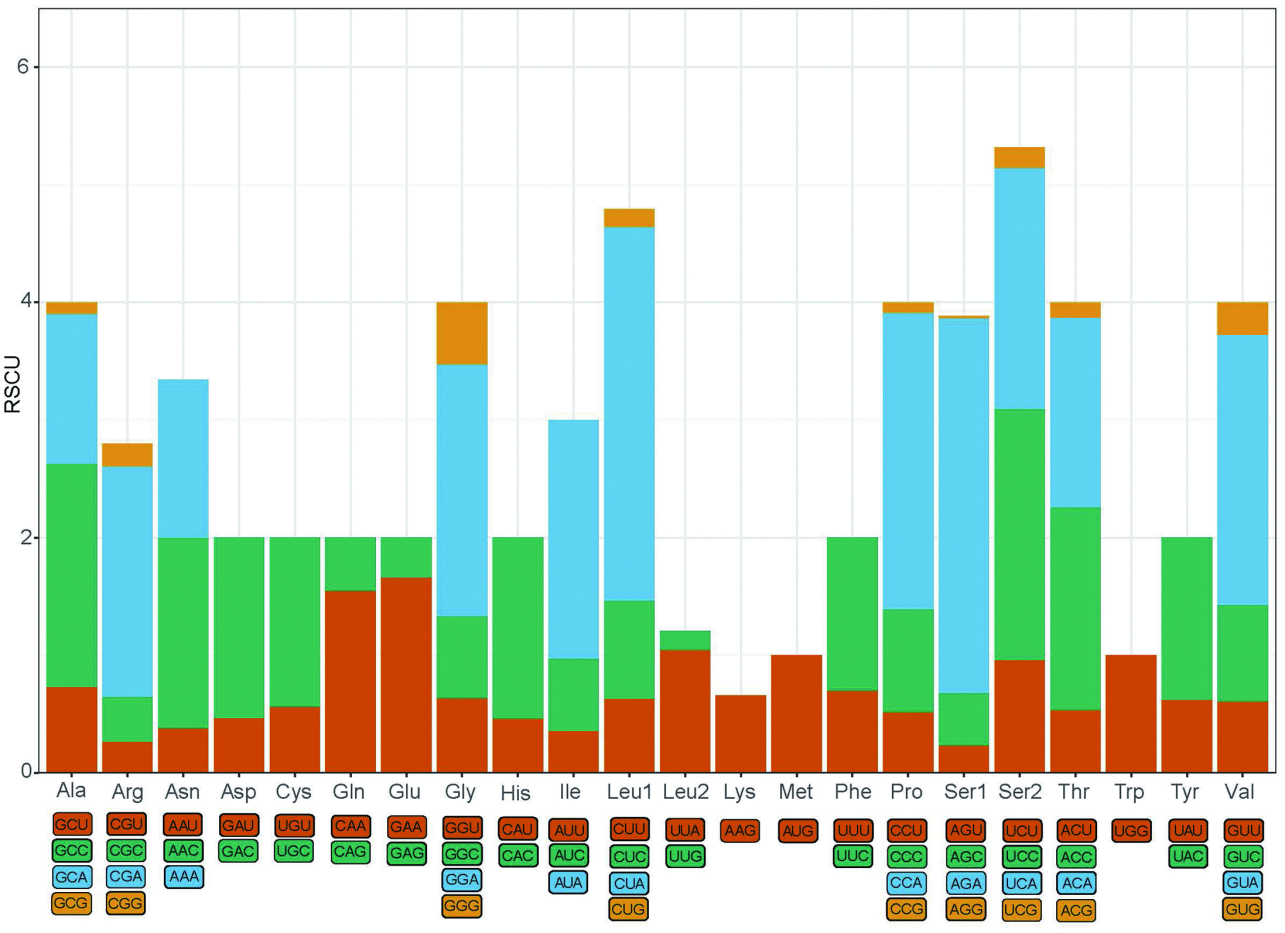
RSCU of *H. atra* mitochondrial PCGs.

From the perspective of amino acid composition, leucine (Leu) is the most abundantly encoded amino acid in the mitochondrial genome of *H. atra*, comprising 16.58% of the total. It is followed by isoleucine (Ile), which accounts for 10.60%. Conversely, cysteine (Cys) and lysine (Lys) are the least encoded amino acids, representing 1.03% and 1.48%, respectively (Fig 3). The mitochondrial genome of *H. atra* contains 61 codons that encode proteins corresponding to 20 amino acids. The frequency distribution chart of codons reveals that the most frequently used codons are AGA (Ser1), CUA (Leu1), and CCA (Pro), while those with relatively lower usage are GCG (Ala), CCG (Pro), and AGG (Ser1). Notably, the third position of the codon predominantly features the A base, with all relative usage frequency values of these codons exceeding 1, indicating a marked preference for this base (Fig 4).

### 3.5 Genetic diversity analysis

Through sequence assembly analysis, we obtained a 625 bp COⅠ gene fragment located in the 250 bp to 874 bp region of the mitochondrial genome and 784 bp COⅠ gene fragment located in the 15010 bp to 15493 bp region of the mitochondrial genome for genetic diversity analysis. The nucleotide genetic diversity parameters for 16S rRNA and COⅠ among the *H. atra* are presented in Table 3. Analysis of the 30 individual 16S rRNA sequences revealed 3 polymorphic loci and 4 distinct haplotypes. The 16S rRNA gene exhibited moderately high haplotype diversity (*H*_*d*_ = 0.697) but low nucleotide diversity (*P*_*i*_ = 0.0026) and low average nucleotide differences (k = 1.234) The genetic distance within this population was found to be 0.003. In contrast, the COⅠ gene showed moderate haplotype diversity (*H*_*d*_ = 0.384), moderate nucleotide diversity (*P*_*i*_ = 0.0072), and moderate nucleotide differences (k = 4.469). The analysis of 30 individual COⅠ sequences identified 13 polymorphic loci and 3 haplotypes. This phenomenon of numerous polymorphic loci is caused by the existence of shared mutations among multiple individuals. The genetic distance within this population was observed to be 0.007. The genetic diversity analysis revealed that the *H. atra* population collected from the coast of Kiribati in this study had a higher genetic diversity compared to the *A. japonicus* from the Sanriku coast and the *H. edulis* from Okinawa Island [25,26].

**Table 3:** The genetic diversity indices of partial 16S rRNA and COⅠ of *H. atra.*

Gene fragment	16S rRNA	COⅠ
Sample number	30	30
Number of haplotye (*H*)	4	3
Haplotye diversity (*H*_*d*_)	0.697	0.384
Number of polymorphic (*S*)	3	13
Average number of nucleotide difference (*k*)	1.234	4.469
Nucleotide diversity (*P*_*i*_)	0.0026	0.0072
Distance within group	0.003	0.007

### 3.6 Phylogenetic relationships analysis

**Fig 5:**
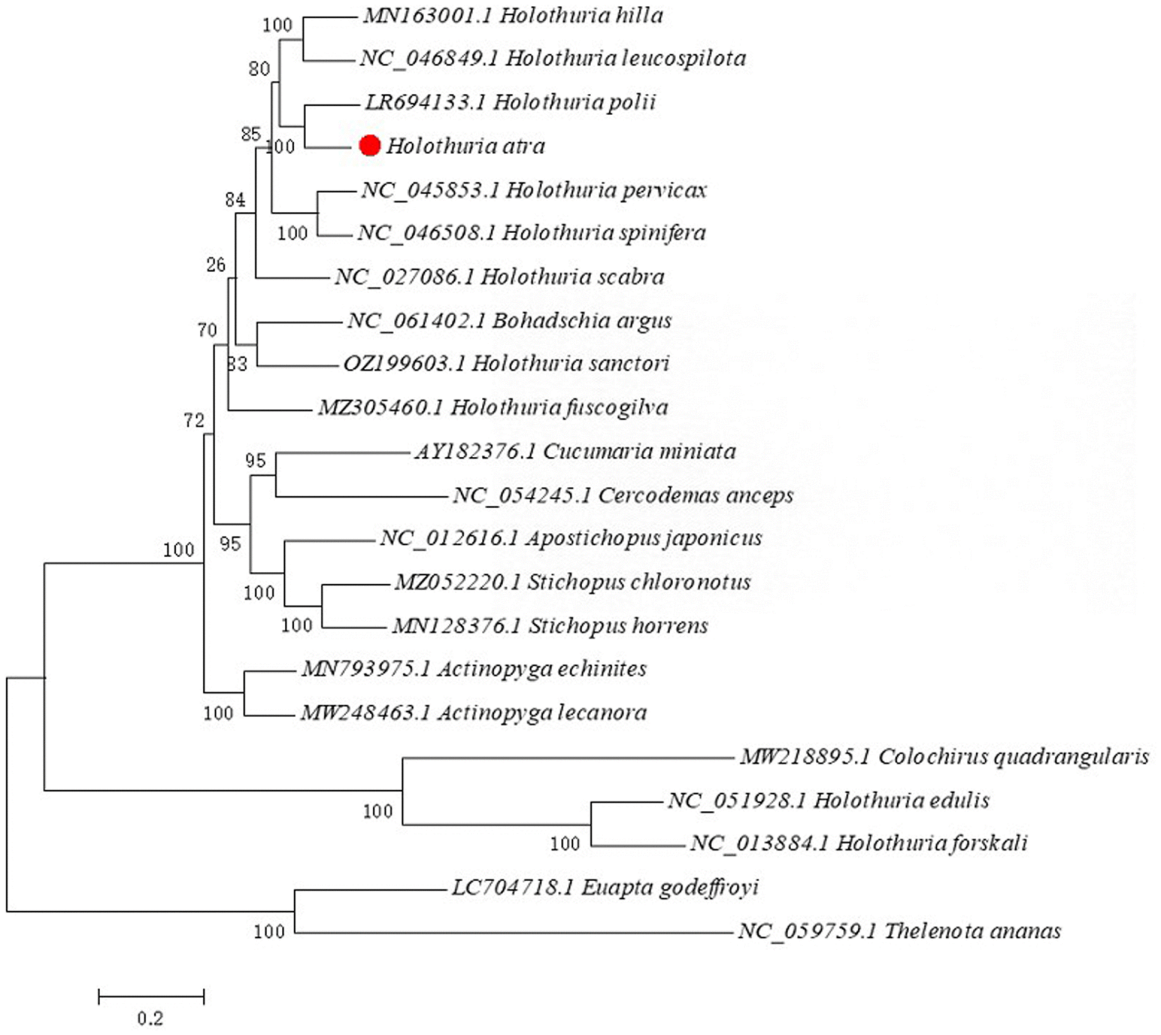
Maximum likelihood phylogenetic tree for the complete mtDNA sequence of Holothuroidea. The position of *H. atra* in the tree is marked by a red circle.

An evolutionary analysis of 22 species within the Holothuroidea (Fig 5) indicates that *H. atra* is most genetically similar to *H. polii*, subsequently clustering with other species in the Holothuriidae. In contrast, *A. echinites* from the Holothuriidae *Actinopyga* groups with *A. lecanora*, forming a distinct branch separate from other Holothuriidae species and those in the Stichopodidae. This finding suggests a significant genetic divergence from other Holothuriidae species. Furthermore, *C. miniata* from the Cucumariidae clusters with *C. anceps*, while *A. japonicus* clusters with *S. chloronotus* and *S. horrens* from the Stichopodidae. Collectively, these two families cluster together, exhibit a relatively close genetic relationship.

### 3.7 Mitochondrial gene order and rearrangements

**Fig 6:**
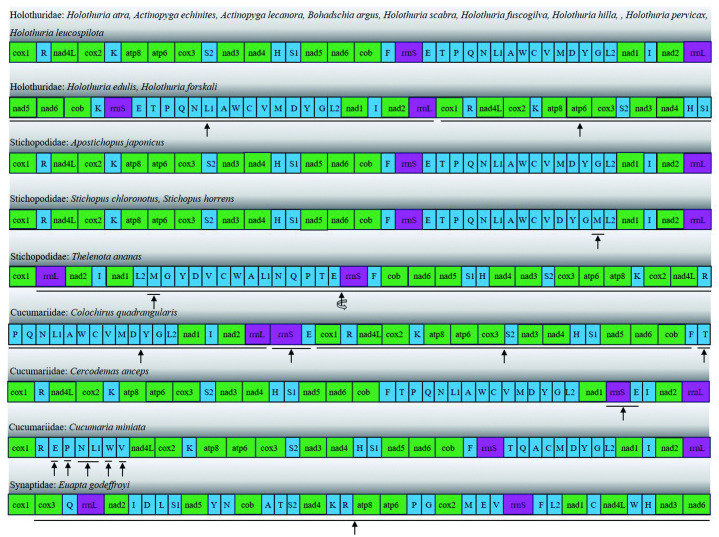
Mitochondrial gene rearrangements of 19 species in Holothuroidea. 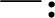
There are regions with gene rearrangement. Short straight lines indicate a gene rearrangement, while long straight lines represent the overall positional changes of multiple genes within the linearly marked area; 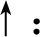
The position undergoes changes before and after; 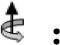
The position has undergone a reversed change in its existence; The meanings of different colors in the figure: green: protein-coding genes, violet: rRNA, blue: tRNA.

By comparing the mitochondrial genome arrangements of 19 different species within the Holothuroidea (Fig 6), it was observed that multiple species exhibited mitochondrial gene rearrangement. This phenomenon was evident not only among species at the order level but also at the family level. For instance, the mitochondrial gene arrangement of nine species in the Holothuriidae, including *H. atra*, differed from that of *H. edulis* and *H. forskali*, yet bore similarities to that of *A. japonicus* from the Stichopodidae. The observed gene rearrangements included alterations in the position of single gene (e.g., the translocation of trnM in *S. chloronotus* and *S. horrens*), shifts in the positions of multiple genes (e.g., in *C. quadrangularis*), and inversions of multi-gene sequences (e.g., in *T. ananas*, where the entire gene sequence was inverted except for cox1). In *E. godeffroyi*, only the cox1 gene maintained the same arrangement as in other species, while all other genes displayed completely altered orders.

## 4. Discussion

In this study, we identified and analyzed the mitochondrial genome composition of *H. atra*. The total length of the mitogenome is 15,788 base pairs (bp), and its organization is comparable to that of the members of other Holothuriidae [27,28]. Mitochondrial PCGs in *H. atra*, like those in other sea cucumbers, typically comprise 13 PCGs, a relatively conserved feature, two rRNAs and 22 tRNAs molecules [24–31]. The AT content of 59.2% indicates a preference for AT base pairs, which is consistent with observations in other Holothuroidea and Echinoidea species [32,33]. The AT-skew value for *H. atra*, measured at 0.154, aligns with the positive values reported for other echinoderms. Most species within the Holothuriidae display positive AT-skew values, suggesting a higher frequency of adenine (A) compared to thymine (T). This phenomenon may be attributed to shared environmental stresses experienced during evolution, which could influence mitochondrial DNA transcription or replication [34,35]. In the PCGs of *H. atra*, the AT-skew values are primarily positive, with the exception of nad6, which shows a negative value of −0.530. Among the rRNA sequences analyzed, all AT-skew values are positive. Of the 22 tRNAs examined, only six—trnS2, trnQ, trnL1, trnA, trnV, and trnD exhibit negative AT-skew values, while the remaining 14 tRNAs show positive AT-skew values.

The mitochondrial genome of *H. atra* encodes 20 distinct amino acids, with Leu being the most abundant, comprising 16.58% of the total, while Cys is the least abundant at 1.03%. This codon usage bias is also evident in related species such as *B. marianensis* and *Stichopus naso* [13,36]. In terms of codon usage frequency, the most frequently utilized codons in the mitochondrial genome of *H. atra* are AGA (Ser1), CUA (Leu1), and CCA (Pro), whereas codons such as GCG (Ala), CCG (Pro), and AGG (Ser1) are utilized less frequently. All codons ending with adenine (A) exhibit RSCU values greater than 1, indicating a preference for A at the third codon position in the mitochondrial genome of *H. atra*. Likewise, species such as *S. naso* and *B. argus* also demonstrate relatively high frequencies of codons ending with A [14,36].

The analysis of rearrangement events within mitochondrial genomes is valuable for phylogeographic and phylogenetic studies, a fact substantiated by numerous studies on vertebrates. Generally, the arrangement of mitochondrial genomes remains relatively stable among vertebrates, including fish, amphibians, and most mammals [37–40]. In contrast, varying degrees of gene recombination are frequently observed in the mitochondrial genomes of invertebrates [28,41]. The same genetic arrangement implies the existence of a common ancestor. Therefore, the analysis of genetic rearrangement is of great significance for the evolutionary relationship analysis of echinoderms. In previous studies, Ma et al. compared the mitochondrial gene arrangements among 20 sea cucumber species, identifying two distinct arrangements [14]. Mu et al. analyzed the mitochondrial gene arrangements of 13 sea cucumber species, revealing seven distinct types [42]. In the present study, we identified eight different types of mitochondrial gene arrangements across 19 species from four families in the Holothuroidea. Notably, two distinct mitochondrial gene arrangements were observed in both the Holothuriidae and Stichopodidae, while three distinct arrangements were found in the Cucumariidae，this indicates that even among different species within the same family, there are significant differences in the arrangement of their genes.

## 5. Conclusion

In summary, this study presents a complete nucleotide sequence of the *H. atra* mitogenome. This research addresses a significant gap in the molecular biology studies of *H. atra*, enhances the mitochondrial genome database for this species, and provides molecular evidence and theoretical references for the classification, identification, and evaluation of germplasm resources of *H. atra*. Furthermore, it lays a foundational data basis for the conservation and sustainable utilization of *H. atra* resources in the Kiribati region.

## Supporting information

S1 Table Codon Table 9 of echinoderms and flatworms. (XLSX)

S2 Table Fig 3 raw data. (XLS)
